# Immune-Mediated Necrotizing Myopathy Manifesting after Five Years of Statin Therapy

**DOI:** 10.1155/2023/1178035

**Published:** 2023-04-24

**Authors:** Nathan G. DeRon, Francis Fischer, Dylan Lopez, Elizabeth C. Brewer

**Affiliations:** ^1^Department of Internal Medicine, Methodist Dallas Medical Center, Dallas, TX, USA; ^2^Department of Internal Medicine, Golden Cross Academic Clinic, Dallas, TX, USA

## Abstract

Immune-mediated necrotizing myopathy (IMNM) is an increasingly common and serious condition in which autoantibodies attack muscle fibers causing clinically significant muscle weakness, fatigue, and myalgias. Recognizing the clinical presentation of IMNM is difficult but necessary, as rapid intervention decreases morbidity. We present a case of a 53-year-old female with IMNM induced by statin therapy with confirmed anti-3-hydroxy-3-methylglutaryl coenzyme A reductase antibodies present on serologic testing. The patient's statin therapy was halted, and the patient was provided with one dose of methylprednisolone and ongoing therapy with mycophenolate. She showed subsequent slow improvements in her muscle weakness and myalgias. It is important for clinicians to be aware of the possible consequences of statin therapy, as these drugs are generally regarded as benign in the medical community. Clinicians should also be aware that statin-induced myopathy can occur at any time during statin therapy. The condition does not necessarily correlate with beginning a new statin medication, as demonstrated in this case in which the patient was on chronic statin therapy before developing symptoms. Continued clinician education and building the fund of medical knowledge regarding this disease are vital to enable clinicians to recognize this disease and act promptly to reduce patient morbidity and improve outcomes.

## 1. Introduction

Immune-mediated necrotizing myopathy (IMNM) is an increasingly common and serious condition in which autoantibodies attack muscle fibers causing clinically significant muscle weakness, fatigue, and myalgia [1–3]. IMNM can be classified into three subtypes: antisignal recognition particles, anti-3-hydroxy-3-methylglutaryl-coenzyme A reductase (anti-HMGCR), and seronegative [4]. Recognizing the clinical presentation of IMNM is difficult but necessary, as rapid intervention decreases morbidity of this disease [5]. IMNM is commonly treated acutely with corticosteroids with add-on therapy of antimetabolites, intravenous immunoglobulins (IVIG), or rituximab for persistent, severe, or therapy-resistant cases [1]. There are no randomized clinical trials helping to guide management of IMNM; therefore, it is important to continue to add to the medical fund of knowledge on this topic with the goal of educating clinicians to recognize and promptly treat this serious disease.

## 2. Case Presentation

A 53-year-old female with a history of type 2 diabetes mellitus, hypertension, and hyperlipidemia presented to the ambulatory clinic with the chief complaint of generalized weakness. The weakness was most profound in the proximal upper and lower extremities. The patient specifically reported no longer being able to lift and carry her grandchild or lift her leg high enough to enter her bathtub. The patient denied other symptoms such as fever, chills, rash, and urinary symptoms. She reported compliance with her current medications, including atorvastatin 20 mg daily, which she had taken for approximately five years. She denied any recent medication changes or significant social history. A review of systems was negative other than the new-onset weakness. Upon physical exam, the patient exhibited 4/5 muscle strength in the bilateral deltoids and hip flexors with 5/5 strength in the biceps, triceps, and gastrocnemius muscles bilaterally. The patient reported mild discomfort with palpation of the skeletal muscle throughout her upper and lower extremities.

Initial laboratory findings most notably revealed markedly elevated creatine kinase (CK), CK-myocardial band, and aminotransferases (Table 1). The patient's atorvastatin was discontinued. Further work-up was ordered, and the patient was referred to rheumatology for further evaluation and management. Follow-up labs illustrated positive anti-HMGCR and anti-Smith/ribonucleoprotein antibody titers (Table 2).

**Figure 1:**
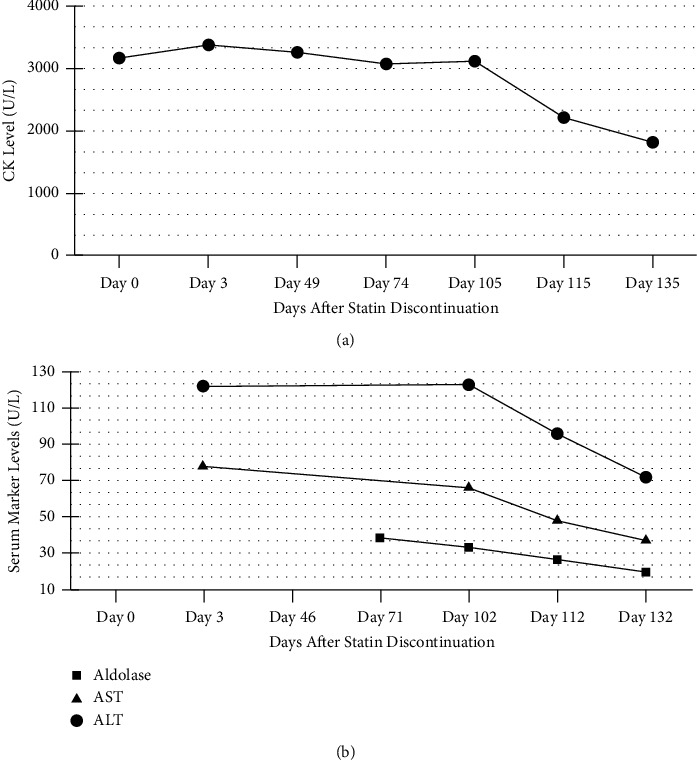
(a) Trend of creatine kinase levels over time after discontinuing statin therapy. (b) Trend of aldolase and aminotransferase levels over time after discontinuing statin therapy.

The patient was diagnosed with IMNM associated with statin therapy. Rheumatology administered one dose of 40 mg of intramuscular methylprednisolone to help with symptoms. More labs were remarkable for normal erythrocyte sedimentation rate, normal C-reactive protein, and elevated aldolase. The patient was started on mycophenolate at a dose of 500 mg twice daily. After one month, this was titrated up to 2500 mg daily split between morning and evening doses. Based on shared decision-making with the patient, glucocorticoid therapy was deferred. The patient's CK, aldolase, and aminotransferase levels were monitored over time. Over a five-month period, the patient's serum CK level showed a progressive reduction (Figure 1(a)). The patient's aldolase, aspartate aminotransaminase, and alanine aminotransferase levels also showed a progressive reduction (Figure 1(b)).

At last follow-up, the patient reported improvement in overall muscle weakness and continued cessation of statin therapy.

## 3. Discussion

Several observational studies estimated an incidence of muscle-related side effects of statin medications at approximately 10% to 15%, although the vast majority of these cases were mild and abated with time while modified statin therapy is continued [6]. IMNM is an increasingly common type of nonhereditary drug-induced myopathy and is driven by sensitization to self-antigens, production of autoantibodies against HMGCR, and to erroneous activation of the complement pathway [2, 6–8]. Patients most often present with proximal muscle weakness, myalgias, and fatigue, but other symptoms such as dysphagia and respiratory failure have been reported [9]. Proximal muscle weakness often includes the posterior thigh, the medial thigh, and the gluteal compartments [10]. Skin involvement has been reported in cases with antibody positivity, but it is a rare clinical manifestation [11]. Risk factors include statin use, especially in cases of anti-HMGCR seropositive IMNM. Atorvastatin was found to be the most frequent offender worldwide. It is unclear if this observation is due to the specific pharmacokinetics of the drug, as it is oxidized by cytochrome p450 3A4 [12], or its increased usage rate compared to other statins. However, there is no data to support that the incidence of IMNM increases with higher statin dosing [13].

IMNM is associated with other medical conditions, including gastrointestinal adenocarcinoma and esophageal, breast, uterine, and ovarian cancers [10, 14–18]. However, increased incidence of cancer in these patients may be associated with increased screening rates given their consistent interaction with healthcare providers. In addition to statin use, risk factors for IMNM include genetic markers such as HLA-DRB1*∗*11:01 and HLA-DRB1*∗*07:01 alleles [10]. Also, statins naturally occur in several types of wild mushrooms, which may serve as a source of statin through ingestion and become a risk factor for IMNM in individuals who commonly consume them [10].

Common laboratory findings almost always include elevated CK, with levels often at least 10 times the upper limit of normal [19]. Other common laboratory findings may include myoglobinuria and elevated serum aminotransferase levels both due to muscle breakdown. Magnetic resonance imaging often reveals evidence of edema in the proximal muscle groups, especially in the lower extremities. These findings are often more severe in patients with seropositive IMNM [20]. There is some recent controversy over the need for invasive muscle biopsy in seropositive IMNM patients because a diagnosis can often be made in the correct clinical context with documented statin use and detection of anti-HMGCR autoantibodies congruent with a significantly elevated CK level [21]. In fact, muscle biopsies often reveal necrosis and muscle regeneration with mild or absent inflammatory infiltrates and inconsistent deposition of complement factors, leading to a significant portion of inconclusive muscle biopsies [22]. Therefore, clinical presentation and laboratory data should be the primary driver of an IMNM diagnosis, with muscle biopsy acting as an additional piece of evidence when the diagnosis remains unclear.

Historically, the cornerstone of therapy for IMNM has been immunomodulators such as corticosteroids in addition to IVIG [23, 24]. However, more recent data shows systemic glucocorticoids, azathioprine, and methotrexate are effective at reducing disease burden and inducing remission with or without the addition of IVIG [25]. In patients who experience relapsing disease during steroid taper, the addition of azathioprine promotes continued disease remission [26]. The combination of glucocorticoids and azathioprine has even been shown to successfully induce remission in seronegative IMNM patients [6]. Additional glucocorticoid-sparing agents such as rituximab and mycophenolate have also been shown to reduce disease burden [19, 27].

## 4. Conclusion

It is important for clinicians to be aware of the possible side effects of statin therapy, as they are generally regarded as benign and rare. Clinicians should also be aware that statin-induced myopathy can occur at any point during statin therapy and does not necessarily correlate with the beginning of statin therapy, as demonstrated in this case. Continued clinician education and building the fund of medical knowledge regarding this disease will enable clinicians to recognize this disease and quickly intervene to reduce patient morbidity and improve outcomes.

## Figures and Tables

**Table 1:** Initial laboratory values.

Lab	Values	Reference range
WBC	7.7 k/*µ*L	3.5–11.0 k/*µ*L
Hemoglobin	14 g/dL	11.5–15.5 g/dL
Hematocrit	43.2%	34.0%–45.0%
Platelets	232 k/*µ*L	130–400 k/*µ*L
Sodium	144 mEq/L	133–146 mEq/L
Potassium	4.3 mEq/L	3.5–5.4 mEq/L
Chloride	105 mEq/L	95–107 mEq/L
Bicarbonate	24 mEq/L	19–31 mEq/L
BUN	12 mg/dL	6–20 mg/dL
Creatinine	0.37 mg/dL	0.6–1.3 mg/dL
AST	78 U/L	9–40 U/L
ALT	122 U/L	5–40 U/L
ALP	84 U/L	40–133 U/L
CK	3164 U/L	28–176 U/L
CKMB	110.0 ng/mL	≤4.3 ng/mL

ALP, alkaline phosphatase; ALT, alanine aminotransferase; AST, aspartate aminotransferase; BUN, blood urea nitrogen; CK, creatine kinase; CKMB, creatine kinase-myocardial band; WBC, white blood cells.

**Table 2:** Follow-up laboratory values.

Lab	Values
Anti-HMGCR level	101 U (strongly positive)
ANA level	1.087 U (slightly positive)
NXP-2 antibody	Negative
TIF1-*γ* antibody	Negative
P155/140 antibody	Negative
PL-7 antibody	Negative
EJ antibody	Negative
Jo-1 antibody	Negative
Smith/RNP antibody	69 U (moderately positive)
SSA 52 antibody	Negative
Fibrillarin antibody	Negative
CCP antibody	Negative
SAE1 antibody	Negative
ANA titer	Negative
MDA5 antibody	Negative
MI-2 antibody	Negative
PL-12 antibody	Negative
OJ antibody	Negative
SRP antibody	Negative
Ku antibody	Negative
PM/Scl 100 antibody	Negative
SSA 60 antibody	Negative
Rheumatoid factor	Negative
CK	3369 U/L

ANA, antinuclear antibodies; CK, creatine kinase; HMGCR, 3-hydroxy-3-methyl-glutaryl-coenzyme A reductase.
